# The Association of Statin Therapy with Liver and Pancreatic Fat Fraction in Type 2 Diabetes Mellitus

**DOI:** 10.3390/diagnostics15040426

**Published:** 2025-02-10

**Authors:** Mehmet Akif Parlar, Hakan Mutlu, Betül Doğantekin, İsmail Serhat Musaoğlu, Nisa Demirboşnak Albayrakoğlu, Mustafa Lütfi Yavuz, Zehra Buşra Özbolat, Mustafa Kaplan

**Affiliations:** 1 Department of Internal Medicine, Sultan 2. Abdülhamid Han Training and Research Hospital, University of Health Sciences, Selimiye Neighborhood, Tıbbiye Street, 34668 Istanbul, Turkey; hakanmutlu1@yahoo.com (H.M.); drbetuldogantekin@gmail.com (B.D.); i.s.musaoglu@gmail.com (İ.S.M.); ndbosnak@hotmail.com (N.D.A.); dr_mustafakaplan@yahoo.com (M.K.); 2 Department of Cardiology, Istanbul University Faculty of Medicine, 34093 Istanbul, Turkey; mustafayavuz135@gmail.com; 3 Deparment of Chest Diseases, Çerkezköy State Hospital, Tekirdağ Provincial Health Directorate, 59100 Tekirdağ, Turkey; dr.zehrabusra@gmail.com

**Keywords:** diabetes mellitus, fatty pancreas, fatty liver, statin, steatosis

## Abstract

**Background/Objectives**: It has been shown that the use of statins in patients with type 2 diabetes mellitus (T2DM) worsens hyperglycemia and hemoglobin A1c levels but may help in the preservation of pancreatic β-cell function. The potential role of a high pancreatic fat fraction (PFF) in this process has not yet been clarified. This study aimed to investigate whether the liver fat fraction (LFF) and PFF in T2DM patients is affected by statin therapy. **Methods**: This cross-sectional study involved a total of 140 T2DM patients, including both those who were receiving (n = 70) and those who were not receiving (n = 70) statin therapy. The mapping of the LFF and PFF utilizing the IDEAL-IQ sequence was conducted in magnetic resonance imaging. **Results**: In T2DM patients who used statins, the median PFF was higher compared to those who did not use statins (8.4 vs. 6.2%, *p* = 0.021), while the median LFF was found to be similar (8.4 vs. 8.9, *p* = 0.572). Variations in PFF were associated with the use of various statins (non-statin group: 6.2 vs. atovastatin: 8.7 vs. rosuvastatin: 3.2 vs. pitavastatin: 9.2, *p* = 0.004). The multivariable regression analysis indicated that insulin usage decreased log(LFF) by a factor of 0.16-fold (ꞵ ± SE = −0.16 ± 0.05, *p* = 0.010), and rosuvastatin usage reduced log(PFF) by 0.16-fold (ꞵ ± SE = −0.16 ± 0.07, *p* = 0.025), irrespective of other risk factors. Furthermore, the use of atorvastatin (ꞵ ± SE = 0.17 ± 0.06, *p* = 0.011) and pitavastatin (ꞵ ± SE = 0.19 ± 0.07, *p* = 0.008) were independently associated with an increase in log(PFF). **Conclusions**: In patients with T2DM, statin use did not show a significant effect on the liver fat fraction, but it caused differences in the pancreatic fat fraction. The observation of a lower pancreatic fat fraction in patients taking a rosuvastatin and atorvastatin dose of 40 mg/day suggests that different types and doses of statins may have varying effects on pancreatic fat accumulation.

## 1. Introduction

Diabetes has a global prevalence exceeding 10%, and it is expected to rise to 12.2% by 2045. Among all diabetes cases, type 2 diabetes mellitus (T2DM) constitutes the majority [1]. Patients with T2DM are at an elevated risk for complications such as cardiovascular disease, kidney damage, neuropathy, and retinopathy, which impose a significant burden on healthcare systems [2]. Notably, recent evidence suggests that even in individuals without diabetes, subclinical glycemic fluctuations leading to increased glycated hemoglobin (HbA1c) levels are associated with subclinical cardiovascular dysfunction [3]. This heightened risk is largely attributed to the presence of metabolic syndrome (MetS), a cluster of interrelated metabolic abnormalities that includes central obesity, insulin resistance, dyslipidemia, and hypertension [4]. While obesity is a well-known risk factor for T2DM, visceral fat has been identified as a stronger determinant of metabolic dysfunction than subcutaneous fat [5,6]. One of the most prominent manifestations of MetS is metabolic dysfunction-associated fatty liver disease (MAFLD), formerly known as non-alcoholic fatty liver disease (NAFLD) [7,8,9]. MAFLD is now recognized as a distinct clinical entity defined by hepatic fat accumulation in the presence of metabolic dysfunction, irrespective of other etiologies such as alcohol consumption [9]. The prevalence of MAFLD is particularly high among individuals with T2DM, and it has been strongly linked to both hepatic and systemic insulin resistance, contributing to the progression of diabetes [10].

In healthy individuals, fatty acids are primarily stored as triacylglycerol in adipose tissue. However, when the storage capacity of adipose tissue is exceeded, fat accumulates in non-adipose tissues, such as the pancreas, liver, and heart [11,12]. This ectopic fat deposition can lead to cellular dysfunction, impair pancreatic β-cell function, contribute to insulin resistance, and ultimately result in elevated blood glucose levels [13]. Moreover, ectopic fat depots are metabolically active and secrete pro-inflammatory cytokines, which further exacerbate insulin resistance and β-cell dysfunction through chronic low-grade inflammation [14]. Increasing evidence suggests a strong association between pancreatic fat accumulation and the onset of both prediabetes and diabetes [15,16]. Since the pancreas and liver play central roles in glucose metabolism, fat deposition in these organs is believed to be a key factor in the pathophysiology of T2DM, particularly through mechanisms related to β-cell dysfunction and insulin resistance [17]. Several studies have shown that patients with T2DM exhibit increased pancreatic fat content and a smaller pancreatic volume compared to healthy individuals [18]. Furthermore, pancreatic fat accumulation has been inversely associated with insulin secretion, indicating its potential role in the progression of diabetes [19,20]. Similarly, hepatic fat accumulation has been strongly linked to insulin resistance during gluconeogenesis and is recognized as a major contributor to the progression of T2DM [21]. A key mechanism underlying β-cell dysfunction in T2DM is glucolipotoxicity, which refers to the combined detrimental effects of chronic hyperglycemia and elevated free fatty acids on pancreatic β-cells. This phenomenon leads to impaired insulin secretion and β-cell apoptosis, thereby exacerbating hyperglycemia and contributing to the progression of diabetes [22,23].

Despite these findings, the association between elevated pancreatic fat content and lipid accumulation or dyslipidemia in diabetic patients remains controversial. While some studies have reported an independent association between fatty pancreas and diabetes without a clear link to dyslipidemia [24,25,26], others have suggested that prediabetic or diabetic patients with a pancreatic fat accumulation are more susceptible to a disrupted lipid metabolism [26,27,28]. Additionally, a positive relationship has been identified between fatty pancreas and elevated lipid levels [29]. Statins, commonly prescribed for diabetic patients with MetS and at cardiovascular risk, are known for their lipid-lowering effects [30]. Evidence from both experimental and clinical studies indicates that statins possess a direct anti-inflammatory effect on adipose tissue [31,32,33]. It has been demonstrated that statins significantly contribute to the improvement of fatty pancreas in rats stimulated with a diet rich in fructose and cholesterol [34]. Although there is limited evidence in the literature specifically addressing the effects of statin therapy on pancreatic or hepatic fat accumulation, studies on other forms of ectopic fat deposition provide supporting insights. A study conducted in patients with aortic stenosis identified a strong correlation between statin therapy and a decreased accumulation of epicardial adipose tissue. Additionally, atorvastatin was reported to exhibit an in vitro anti-inflammatory effect on cultured epicardial adipose tissue adipocytes [33]. Furthermore, another study demonstrated that statins reduced epicardial adipose tissue attenuation on computed tomography, independent of their lipid-lowering effects. This suggests that statins may decrease the metabolic activity of epicardial fat through mechanisms involving reduced cellularity, vascularity, or inflammation [35]. These findings highlight the broader pleiotropic effects of statins on ectopic fat depots, warranting further investigation into their potential impact on pancreatic fat accumulation and metabolism.

Given the established significance of ectopic fat accumulation in metabolic dysregulation, whether statin therapy could favorably influence pancreatic fat in humans with T2DM remains unclear. While preclinical models suggest that statins can ameliorate fatty pancreas [34], and clinical studies demonstrate the beneficial effects of statins on other ectopic fat depots such as epicardial adipose tissue [33,35], there is a distinct lack of direct human data examining statin use and the pancreatic fat fraction in T2DM. This gap is especially pertinent given the central role of pancreatic β-cell function in diabetes pathophysiology and the potential for targeted interventions to improve clinical outcomes. Moreover, given the observed effects of statins on epicardial adipose tissue, it is plausible that similar mechanisms could extend to pancreatic and hepatic fat depots.

We hypothesized that statin therapy may be associated with a lower pancreatic fat fraction in patients with T2DM, potentially through mechanisms involving reduced inflammation and adipose tissue remodeling. Therefore, this study aimed to investigate whether the pancreatic fat fraction in patients with T2DM is affected by statin therapy. By addressing this research gap, our study aims to provide new insights into the metabolic effects of statins beyond their conventional lipid-lowering role.

## 2. Materials and Methods

This cross-sectional study was conducted on T2DM patients under outpatient follow-up at the Internal Medicine Clinic of Sultan Abdülhamid Han Training and Research Hospital, between December 2021 and December 2022. The study was approved by the local ethics committee (date: 9 February 2022, decision No: 101412) and was carried out in accordance with the relevant ethical guidelines and the Helsinki Declaration (2013 Brazil revision). All study participants provided written informed consent.

An a priori power analysis was conducted to determine the required sample size for the study, based on pilot data collected from 10 patients in each group. The primary outcome was the difference in pancreatic fat fraction between T2DM patients using statins (10.2 ± 5.7) and those not using statins (7.5 ± 5.1). The effect size for the pancreatic fat ratio was determined to be 0.50 using Cohen’s formula, indicating a moderate effect size. Using the G*Power (version 3.1.9) program, the sample size was determined to be 70 patients per group, with a 90% power and a 5% margin of error.

### 2.1. Study Population

A total of 140 patients with T2DM (77 female [55.0%], 63 male [45.0%]) who had received upper abdominal magnetic resonance imaging (MRI) for different indications (abdominal pain, fatty liver monitoring, etc.) were evaluated. Patients were consecutively recruited from internal medicine clinics. The inclusion criteria required patients to be aged 18–70, diagnosed with T2DM, and to have either been using statin therapy for at least one year prior to the abdominal MRI or to have never used statins, and to have consented to participate in the study. All patients were enrolled from the same clinical environment, where a Mediterranean-style diet is routinely recommended as part of standard diabetes management, and adherence to this diet was included as one of the inclusion criteria. Patients with a history of systemic inflammatory or autoimmune diseases, heart failure, active rheumatologic conditions, malignancy, and pregnancy were excluded from the study. The study analyzed 70 patients with T2DM who were on statin therapy and met the eligibility criteria, along with 70 T2DM patients not on statins, matched for age and gender.

At the time of the patients’ outpatient clinic visits, their demographic information, laboratory results, and prescribed medications were documented. Anthropometric parameters, including height, weight, and waist circumference, were measured by trained healthcare staff in the outpatient clinic using standardized equipment. Body mass index (BMI) was calculated as weight in kilograms divided by height in meters squared (kg/m^2^). Alcohol consumption was assessed as drinking alcohol weekly or more frequently over a minimum period of one year. The patients’ pancreas and liver fat fraction were assessed by an expert radiologist with at least five years of experience, based on abdominal MRI images taken within the last year.

### 2.2. Laboratory Measurements

After a fasting period of 8–10 h, venous blood samples from all patients were obtained and evaluated using the Beckman Coulter LH 780 device (Mervue, Galway, Ireland) and the Hitachi Modular P800 autoanalyzer (Roche Diagnostics Corp., Indianapolis, IN, USA). Levels of hemoglobin (photometric method), leukocytes (impedance method), C-reactive protein (CRP) (immunoturbidimetric method), albumin (bromocresol green method), fasting blood glucose (FBG) (enzymatic colorimetry), glycated hemoglobin (HbA1c) (high-performance liquid chromatography), urea (urease/glutamate dehydrogenase method), creatinine (enzymatic colorimetry, Jaffe method), alanine aminotransferase (ALT) and aspartate aminotransferase (AST) (kinetic UV method), alkaline phosphatase (ALP) (p-Nitrophenyl phosphate method), gamma-glutamyl transferase (GGT) (γ-glutamyl-3-carboxy-4-nitroanilide method), and amylase and lipase (enzymatic colorimetry) were determined. Total cholesterol, high-density lipoprotein cholesterol (HDL-C), and triglyceride levels were measured using enzymatic colorimetry. The Friedewald formula was used to determine low-density lipoprotein cholesterol (LDL-C) [36].

### 2.3. MRI Measurements

Pancreatic MRI imaging was carried out using a 1.5 Tesla scanner (General Electric, 1.5 T GE Signa Artist, Milwaukee, WI, USA), which includes an eight-channel body phase array extremity coil. The MRI protocol comprised conventional 2-plane (coronal and axial) T2-weighted, fat-suppressed axial T2-weighted, diffusion weighted imaging (DWI) and apparent diffusion coefficient (ADC) mapping, a dual-echo T1 in phase and out of phase, and iterative decomposition of water and fat with echo asymmetry and least squares estimation (IDEAL-IQ) sequences.

**Figure 1:**
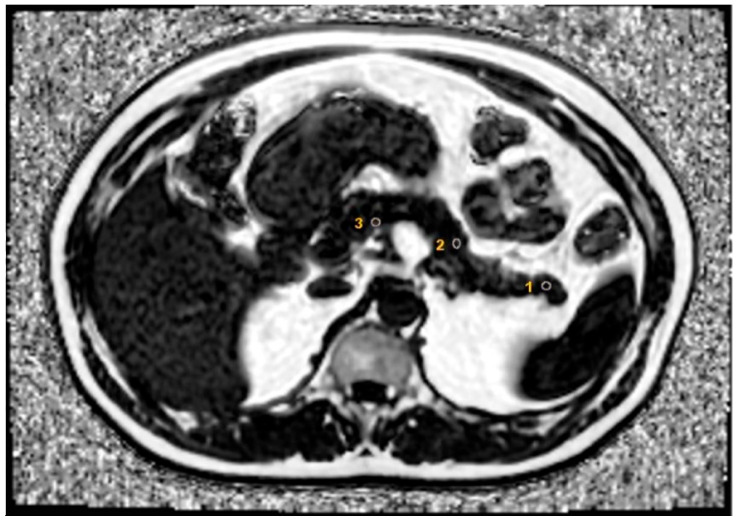
For the use of the IDEAL-IQ fat fraction ratio, the areas are marked in the pancreas. The numbered regions correspond to the tail (1), body (2), and head (3) of the pancreas.

In the MR spectrum, water molecules exhibit a single peak, whereas fat molecules display multiple peaks. The IDEAL-IQ technique includes acquiring images at various and asymmetric echo times to address these multiple fat peaks, enabling T2* corrections based on multiple chemical shifts, independent of T1. In the IDEAL-IQ sequences, the parameters were TR = 35.3 ms, FOV = 36–42 cm, matrix = 128 × 128, bandwidth = 125 kHz, and a slice thickness of 8 mm, with an approximate total of 72–112 slices covering all upper abdominal structures, including the pancreas. The analysis of IDEAL-IQ images was carried out on an imaging workstation (Advantage Workstation VolumeShare7; GE Healthcare, General Electric Company, Milwaukee, WI, USA) by a single experienced radiologist with over five years of expertise in abdominal imaging. For the liver fat percentage analysis, images were captured from eight different segments, carefully excluding major vascular formations. In the pancreas, while excluding the pancreatic duct, two elliptical Regions of Interest (ROIs), each of 15 mm^2^, were placed in the head, body, and tail areas (Figure 1). All calculations have been automatically corrected using homogeneity maps.

### 2.4. Hepatobiliary USG

All patients’ hepatobiliary ultrasound images, taken after a minimum of 8–10 h of fasting, were performed on a Samsung RS85 device (Samsung Medison, Seoul, Republic of Korea), equipped with a Samsung CA1-7A convex probe with a bandwidth of 1–7 MHz. As echogenicity increases in the liver parenchyma due to rising fat levels, the penetration of ultrasound waves diminishes with an increased fat accumulation, making it harder to visualize deeper parts of the parenchyma (such as near the subhepatic–diaphragm area and hepatic vein). Accordingly, the liver steatosis was classified as follows: (1) liver with normal parenchyma; (2) mild hepatosteatosis (Grade I), where the borders of vascular structures can be discerned, with the liver parenchyma appearing the same or more echogenic compared to the spleen parenchyma; (3) moderate hepatosteatosis (Grade II), characterized by increased echogenicity, where the borders of vascular structures cannot be discerned; and (4) severe hepatosteatosis (Grade III), where the borders of vascular structures are indiscernible, there is a marked increase in echogenicity, and deep parenchymal areas like that adjacent to the diaphragm cannot be visualized [37,38,39]. The assessment of patients was stratified according to the presence or absence of hepatic steatosis. The ultrasound evaluations were conducted by a single radiologist with at least five years of experience in hepatobiliary imaging. The evaluation of the pancreas was conducted by describing its echogenicity in comparison to the spleen parenchyma, without making any judgment related to steatosis.

### 2.5. Statistical Analysis

All data were analyzed with IBM SPSS Statistics for Windows 20.0 (IBM Corp., Armonk, NY, USA). Numerical data determined to be normally distributed based on the results of Kolmogorov–Smirnov tests are given as the mean ± standard deviation, while non-normally distributed variables are given as the median (25th–75th quartile). For comparisons between groups, the Student t-test or Mann–Whitney U test and ANOVA test or Kruskall–Wallis H test were used in line with the normality of the considered distribution. Categorical variables are given as numbers and percentages, and inter-group comparisons were conducted with Chi-square and Fisher exact tests. Spearman correlation analyses were applied to evaluate the relationships between numerical variables. A multivariable Cox regression analysis with the backward Wald method was subsequently performed to identify any possible independent predictors of the fat fraction of the liver and pancreas. Significance was accepted at *p* < 0.05 (*) for all statistical analyses.

## 3. Results

The study population included 140 patients with T2DM, of which 70 were statin users (mean age: 57.4 ± 7.5 years) and 70 were non-users of statins (mean age: 55.7 ± 8.7 years). Among the patients using statins, 75.7% (n = 53) were on atorvastatin, 11.4% (n = 8) on rosuvastatin, and 12.9% (n = 9) on pitavastatin. Of those taking atorvastatin, 47.1% (n = 33) were on a dose of 10 mg/day, 34.3% (n = 24) were taking 20 mg/day, and 18.5% (n = 13) were on 40 mg/day. The median duration of statin use was 2 years (IQR range = 1–5 years). The demographic and clinical characteristics of the T2DM patients are shown in Table 1 in detail.

**Figure 2:**
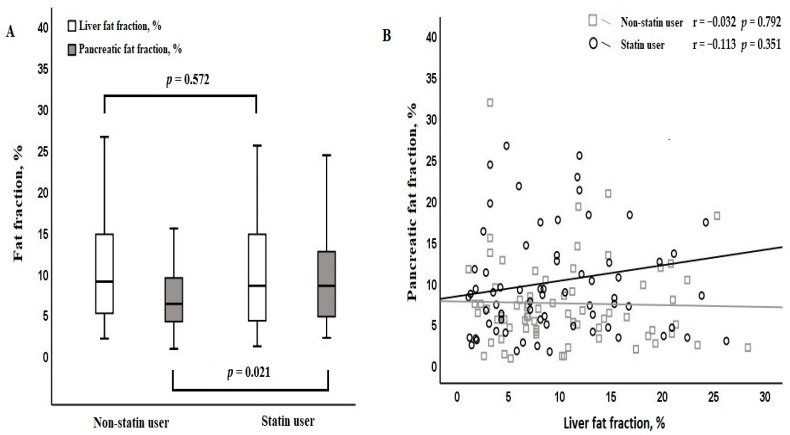
In type 2 diabetes mellitus patients, the distribution of liver and pancreatic fat fractions based on statin usage (**A**) and the correlation existing between these fractions (**B**).

In T2DM patients who used statins, the median duration of T2DM, as well as the rates of hypertension and hyperlipidemia, were higher compared to those who did not use statins. Other demographic characteristics did not show significant differences between the groups. In T2DM patients who used statins, the median pancreatic fat fraction level was higher compared to those who did not use statins (8.4 vs. 6.2%, *p* = 0.021) (Figure 2A), while the median liver fat fraction level was found to be similar (8.4 vs. 8.9, *p* = 0.572) (Figure 2A) (Table 1).

Among the statin subgroups that shared similar demographic features (Supplementary Table S1), the rosuvastatin group exhibited a lower pancreatic fat fraction compared to both the group not using statins and other groups receiving statin treatment. Meanwhile, the atorvastatin and pitavastatin groups showed a comparable pancreatic fat fraction, which was elevated when compared to the non-statin user group (non-statin group: 6.2 vs. atorvastatin: 8.7 vs. rosuvastatin: 3.2 vs. pitavastatin: 9.2, *p* = 0.004) (Table 2).

No correlation was found between liver fat fraction and pancreatic fat fraction in the all population (r = 0.035, *p* = 0.705). Similarly, the use of statins showed no variation in this relationship (non-statin group, r = −0.032, *p* = 0.792; statin group, r = 0.113, *p* = 0.351) (Figure 2B). While a positive correlation existed between BMI and waist circumference and liver fat fraction, they did not show any correlation with the pancreatic fat fraction. No relationship was found between comorbid conditions and either liver fat fraction or pancreatic fat fraction (Table 2). In patients using only insulin, the median liver fat fraction was lower than in those receiving other types of anti-diabetic therapies. Similarly, in the group receiving a combined treatment, the liver fat fraction was lower compared to those taking only OADs (only OADs: 10.5 vs. only insulin: 4.8 vs. combined therapy: 7.4, *p* = 0.032). There were no significant variations in the median pancreatic fat fraction levels among different antidiabetic treatment groups (Table 2).

There was a positive correlation between liver fat fraction and levels of ALT (r = 0.348, *p* < 0.001) and triglycerides (r = 0.458, *p* < 0.001), while a positive correlation existed between pancreatic fat fraction and ALP level (r = 0.484, *p* < 0.001). Other laboratory parameters did not show a correlation with either liver or pancreatic fat fractions (Table 3).

Potential risk factors associated with the liver and pancreatic fat fraction were shown in a univariable linear regression analysis. The multivariable regression analysis indicated that insulin usage decreased the log(liver fat fraction) by a factor of 0.16-fold (ꞵ ± SE = −0.16 ± 0.05, *p* = 0.010), and rosuvastatin usage reduced the log(pancreatic fat fraction) by 0.16-fold (ꞵ ± SE = −0.16 ± 0.07, *p* = 0.025), irrespective of other risk factors. Furthermore, the use of atorvastatin (ꞵ ± SE = 0.17 ± 0.06, *p* = 0.011) and pitavastatin (ꞵ ± SE = 0.19 ± 0.07, *p* = 0.008) were independently associated with an increase in log(pancreatic fat fraction) (Table 4).

## 4. Discussion

To the best of our knowledge, this is the first study investigating the relationship between the pancreatic fat fraction and statin use in patients with T2DM. The main findings of this study were as follows: (1) In statin users, there was an increased pancreatic fat fraction compared to those not using statins, but the liver fat fraction did not exhibit any significant difference. (2) Variations in the pancreatic fat fraction were associated with the use of various statins. (3) There was no correlation between the liver fat fraction and the pancreatic fat fraction.

Growing evidence indicates that the accumulation of fat in the pancreas impacts the metabolism of glucose and lipids [24,25,26,27,28]. Lifestyle changes like diet, weight loss, or insulin resistance management, along with bariatric surgery, can reduce pancreatic fat levels [40]. Innovative approaches such as the microbiota and fecal microbiota transplantation are emerging as promising therapeutic options for pancreatic and liver disease [41,42]. Nevertheless, the majority of studies investigating the impact of drugs on fatty pancreas have been carried out using in vitro methods or mouse models [43]. A study assessing the impact of telmisartan, sitagliptin, or their combination on the ultrastructural alterations in the pancreases of mice fed a high-fat diet found that both the monotherapy and the combined treatment effectively reversed pancreatic steatosis [44]. A study investigating the effect of glucagon-like peptide-1 analogs on ectopic fat accumulation in obese patients with T2DM showed that exenatide effectively reduces liver fat and epicardial fat levels [45]. Another study on patients with T2DM and non-alcoholic fatty liver disease (NAFLD) revealed that 24 weeks of dapagliflozin treatment led to a notable decrease in both liver and pancreatic fat content [46]. Contrary to these findings, there are studies reporting that in prediabetes or T2DM patients, treatment with metformin or empagliflozin had no effect on pancreatic fat content [47,48]. In this study, antidiabetic drugs were not associated with the pancreatic fat fraction, but insulin use was linked to a reduction in the liver fat fraction. These variations among studies might be due to factors like the criteria for choosing patients, the size of the sample groups, and ethnic origins [49].

It has been shown that statins may potentially lead to a decrease in the metabolic activity of adipose tissue [35]. However, research in this area predominantly targets NAFLD and non-alcoholic steatohepatitis (NASH), showing that statins effectively lower liver enzymes and positively influence liver histology in these conditions [50,51,52]. Interestingly, this study showed that in T2DM patients using statins, the pancreatic fat fraction was higher compared to the control group, but it did not cause a significant difference in the liver fat fraction. This condition could be associated with the simultaneous progression of lipid buildup and β-cell dysfunction in the pancreas. A study conducted in rats has shown that lipid accumulation in pancreatic tissue parallels the development of β-cell insufficiency, both in terms of timing and magnitude [53]. Statins inhibit the synthesis of cholesterol through the HMG-CoA reductase pathway, leading to the enhanced entry and buildup of cholesterol in pancreatic β-cells. This process disrupts β-cell functionality through the alteration in glucose-induced Ca2+ signaling pathways [54]. Contrary to these findings, a study assessing pancreatic fat content in subjects with prediabetes, diabetes, and the general population without cardiovascular disease, using magnetic resonance imaging, demonstrated that lipid-lowering drugs had a protective effect in a univariate analysis. However, this significance was lost in a multivariate regression analysis [15]. However, these relationships may be strongly associated with the type and dosage of statins [55].

Glucolipotoxicity, a key mechanism in β-cell dysfunction, refers to the synergistic toxic effects of chronic hyperglycemia and elevated free fatty acids on pancreatic β-cells, ultimately leading to impaired insulin secretion and β-cell apoptosis [22,23]. Recent findings indicate that glucolipotoxicity induces lipid partitioning imbalances, mitochondrial dysfunction, and the accumulation of reactive oxygen species, cholesterol, and ceramides, all of which contribute to β-cell failure [56]. A previous study indicated that lipophilic statins could influence insulin secretion via HMG-CoA inhibition or cytotoxicity, while hydrophilic statins did not have this effect [57]. Subclasses of statins differ in function in terms of oral absorption, bioavailability, hepatic extraction, protein binding, and HMG-CoA reductase activity. Simvastatin, atorvastatin, fluvastatin, and lovastatin, being hydrophobic, undergo metabolism through the cytochrome P450 enzyme system. Conversely, pravastatin and pitavastatin, being hydrophilic, are minimally metabolized in the liver, with rosuvastatin exhibiting a moderately distinct behavior [58]. The research indicates that statins with hydrophobic properties achieve greater concentrations in the liver than hydrophilic statins [59]. Patients with T2DM who used atorvastatin and rosuvastatin showed a tendency towards lower liver fat fractions. However, the pancreatic fat fraction was significantly lower only in the rosuvastatin group compared to the atorvastatin and pitavastatin ones. There are studies indicating that pitavastatin has neutral effects on adipocytes and insulin resistance [60,61]. Moreover, rosuvastatin, which is associated with a lower pancreatic fat fraction, may exhibit a pleiotropic effect by altering the fat distribution from internal organs to subcutaneous fat depots [62]. On the other hand, in patients using a 40 mg dose of atorvastatin, both the liver and pancreatic fat fractions were observed to be lower. An experimental study in obese mice indicated that treatment with atorvastatin (30 mg/kg/day) maintained the functionality of pancreatic β-cells, linked to enhanced pancreatic regeneration and the alleviation of pancreatic ER stress [63]. The current findings support the pleiotropic effect of rosuvastatin on adipose tissues, as well as the dose-dependent effect of atorvastatin. However, contrary findings have also been reported. In another experimental study conducted on Zucker rats, the effect of 6-week treatments with various statins (simvastatin, pravastatin, rosuvastatin, atorvastatin, lovastatin, and fluvastatin) on body and liver fat accumulation was investigated. It was reported that all statins, except simvastatin, worsened insulin resistance. Additionally, significant increases in adipose tissue were observed in rats treated with rosuvastatin, atorvastatin, fluvastatin, and lovastatin [64]. The variations in the existing literature underscore the requirement for additional prospective, randomized controlled trials.

Our study has several strengths. It provides novel insights into the potential impact of statin therapy on pancreatic fat accumulation in patients with T2DM, an area that has not been extensively studied. Additionally, our study utilized magnetic resonance imaging (MRI)-based fat quantification, which is considered one of the most reliable non-invasive methods for assessing pancreatic and hepatic fat content. The inclusion of a well-characterized cohort of T2DM patients with detailed clinical and biochemical assessments further strengthens our findings. Moreover, the use of multivariable regression models to adjust for potential confounders enhances the robustness of our results, allowing for a more nuanced evaluation of the independent associations between statin use and ectopic fat accumulation.

Our study also has several limitations. Firstly, this study was single-centered and had a relatively small sample size, which significantly affected the sample size in the statin subgroups. Secondly, temporal changes in the patients’ lipid profiles and liver and pancreatic fat fractions were not examined. Thirdly, fatty pancreas, a relatively new clinical entity, has not yet had its significance and diagnostic methodologies comprehensively defined, with diagnosis primarily based on an histopathological evaluation. Finally, no evaluation was conducted on additional data related to the patients’ diets or on anthropometric measurements, including muscle and fat assessments.

## 5. Conclusions

In patients with T2DM, statin use did not show a significant effect on the liver fat fraction, but it caused differences in the pancreatic fat fraction. The observation of a lower pancreatic fat fraction in patients taking a rosuvastatin and atorvastatin dose of 40 mg/day suggests that different types and doses of statins may have varying effects on pancreatic fat accumulation. In the management of T2DM, specific statins may guide personalized treatment strategies by modulating pancreatic lipid metabolism.

## Figures and Tables

**Table 1:** Demographic and clinical characteristics of patients with type 2 diabetes mellitus.

Variables	All Population n = 140	Statin Use		*p*
		No	Yes	
		n = 70	n = 70	
Female gender, n (%)	77 (55.0)	40 (57.1)	37 (52.9)	0.610
Age, years	56.5 ± 8.2	55.7 ± 8.7	57.4 ± 7.5	0.225
BMI, kg/m^2^	31.1 ± 7.6	30.9 ± 8.0	31.2 ± 7.3	0.776
Waist circumference, cm	103.4 ± 12.1	102.9 ± 11.4	103.9 ± 12.8	0.637
Alcohol use, n (%)	19 (13.6)	12 (17.1)	7 (10.0)	0.324
Duration of diabetes, years	7.0 (3.2–13.0)	6.0 (2.2–12.0)	8.0 (5.0–15.0)	0.050 *
Comorbidities, n (%)				
Hypertension	65 (46.4)	26 (37.1)	39 (55.7)	0.028 *
Cardiac disease	14 (10.0)	5 (7.1)	9 (12.9)	0.260
Thyroid diseases	16 (11.4)	7 (10.0)	9 (12.9)	0.595
Hyperlipidemia	88 (62.9)	22 (31.4)	66 (94.3)	<0.001 *
Anti-diabetic drugs				
Only OADs	92 (65.7)	46 (65.7)	46 (65.7)	0.684
Only insulin	4 (2.9)	1 (1.4)	3 (4.3)	
Combined therapy	44 (31.4)	23 (32.9)	21 (30.0)	
Hepatosteatosis, n (%)	122 (87.1)	64 (91.4)	58 (82.9)	0.207
Liver fat fraction, %	8.5 (4.5–14.7)	9.0 (5.1–14.7)	8.4 (4.2–14.7)	0.572
Pancreatic fat fraction, %	7.2 (4.4–11.4)	6.2 (4.2–9.3)	8.4 (4.7–12.6)	0.021 *
Laboratory findings				
Glucose, mg/dL	134.5 (107.0–174.5)	142.0 (121.0–184.5)	130.5 (103.0–161.8)	0.060
Leukocytes, 10^3^/mm^3^	7.9 ± 2.2	7.9 ± 2.5	7.8 ± 1.9	0.821
Platelets, 10^3^/mm^3^	271.5 ± 58.5	269.1 ± 58.1	273.9 ± 59.2	0.634
HbA1-c, %	7.5 (6.5–9.0)	7.7 (6.6–9.8)	7.1 (6.4–8.6)	0.073
Urea, mg/dL	30.0 (25.0–34.0)	29.0 (25.0–32.0)	30.0 (25.0–36.0)	0.255
Creatinine, mg/dL	0.8 (0.6–0.9)	0.7 (0.6–0.8)	0.8 (0.7–0.9)	0.035 *
ALT, U/L	18.8 (14.0–25.0)	18.1 (14.0–23.0)	19.0 (14.5–25.0)	0.479
AST, U/L	17.0 (14.0–20.0)	16.0 (14.0–18.8)	17.0 (15.0–20.7)	0.047 *
ALP, U/L	78.0 (65.8–96.0)	77.5 (64.0–96.0)	79.5 (69.0–95.8)	0.298
GGT, U/L	23.0 (16.0–32.0)	23.0 (15.2–30.8)	24.5 (17.0–35.5)	0.216
Amylase, U/L	61.0 (45.5–77.8)	54.0 (41.2–82.8)	63.0 (54.2–74.0)	0.142
Lipase, U/L	30.0 (24.0–41.0)	32.5 (23.0–41.5)	28.5 (24.4–40.5)	0.467
Cholesterol, mg/dL	197.5 ± 58.2	221.6 ± 66.5	180.4 ± 44.8	0.002 *
HDL-C, mg/dL	47.7 ± 12.8	48.4 ± 12.8	46.9 ± 12.9	0.495
LDL-C, mg/dL	110.5 (86.0–140.0)	119.5 (96.2–142.8)	100.0 (78.0–133.8)	0.009 *
Triglycerides, mg/dL	142.0 (97.8–195.8)	164.0 (101.2–212.2)	132.0 (97.2–181.5)	0.087

Numerical variables are shown as mean ± standard deviation or median (IQR). Categorical variables are shown as numbers (%). * *p* < 0.05 shows statistical significance. Abbreviations: ALP, alkaline phosphatase; ALT, alanine aminotransferase; AST, aspartate aminotransferase; BMI, body mass index; GGT, gamma-glutamyl transferase, HbA1-c, hemoglobin A1c; OAD, oral anti-diabetic drug; HDL-C, high-density lipoprotein cholesterol; LDL-C, low-density lipoprotein cholesterol.

**Table 2:** The relationship between demographic characteristics and the fat fractions of the liver and pancreas in patients with type 2 diabetes mellitus.

Variables	LFF (%)		PFF (%)	
	Median (IQR) or Correlation Coefficient (r)	*p*	Median (IQR) or Correlation Coefficient (r)	*p*
Gender				
Female	11.3 (6.7–16.7)	0.002 *	7.0 (4.9–10.3)	0.752
Male	6.9 (3.8–11.3)		7.4 (3.9–12.5)	
Age	−0.003	0.975	0.167	0.149
BMI	0.310	<0.001 *	0.090	0.292
Waist circumference	0.224	0.008 *	0.148	0.082
Alcohol use				
No	9.0 (4.4–14.8)	0.191	7.2 (4.5–11.2)	0.966
Yes	6.1 (4.6–11.2)		7.5 (3.5–11.6)	
Duration of diabetes	−0.045	0.601	0.074	0.385
Hypertension				
No	7.8 (4.3–13.8)	0.253	7.1 (4.3–9.7)	0.240
Yes	9.8 (5.8–14.7)		7.2 (4.5–13.3)	
Cardiac disease				
No	9.2 (4.6–14.8)	0.370	7.2 (4.5–10.6)	0.367
Yes	8.1 (3.8–10.8)		8.3 (4.2–17.3)	
Thyroid diseases				
No	8.5 (4.7–14.7)	0.958	7.3 (4.5–11.0)	0.888
Yes	10.6 (3.8–15.0)		6.6 (4.3–11.5)	
Hyperlipidemia				
No	10.4 (5.5–14.8)	0.509	6.7 (4.5–9.2)	0.112
Yes	8.4 (4.3–14.5)		7.6 (4.4–12.5)	
Anti-diabetic drugs				
Only OADs	10.5 (6.0–15.3) ^†^	0.032 *	7.3 (4.3–11.1)	0.906
Only insulin	4.8 (3.4–7.1) ^†^		7.2 (4.1–9.0)	
Combined therapy	7.4 (4.2–13.2) ^†^		6.9 (4.5–12.0)	
Duration of statin	−0.086	0.315	0.142	0.194
Statin use				
No	9.0 (5.1–14.7)	0.445	6.2 (4.2–9.3) ^†^	0.004 *
Atorvastatin	8.3 (3.8–13.0)		8.7 (5.5–14.9)	
Rosuvastatin	8.1 (5.1–16.1)		3.2 (2.6–6.6) ^†^	
Pitavastatin	14.8 (7.8–20.1)		9.2 (3.5–10.2)	
Dose of atorvastatin				
10 mg/day	8.5 (5.1–13.9)	0.009 *	8.8 (5.4–15.4)	0.042 *
20 mg/day	11.9 (4.4–22.0)		9.0 (5.1–13.5)	
40 mg/day	4.8 (1.6–8.0) ^†^		5.5 (2.8–8.9) ^†^	

* *p* < 0.05 shows statistical significance. ^†^ Indicates groups that showed differences in the post hoc analysis. Abbreviations: BMI, body mass index; IQR, interquartile range; LFF, liver fat fraction; PFF, pancreatic fat fraction; OADs, oral anti-diabetic drugs.

**Table 3:** Relationship between laboratory findings and liver and pancreatic fat fraction in patients with type 2 diabetes mellitus.

Variables	LFF		PFF	
	r	*p*	r	*p*
Glucose	0.168	0.166	0.025	0.836
Leukocytes	0.184	0.130	0.023	0.848
Platelets	0.146	0.232	−0.057	0.644
HbA1-c	0.016	0.899	0.013	0.918
Urea	−0.032	0.796	−0.025	0.838
Creatinine	−0.170	0.161	−0.073	0.553
ALT	0.348	<0.001 *	0.228	0.090
AST	0.220	0.102	0.149	0.223
ALP	0.230	0.080	0.484	<0.001 *
GGT	0.162	0.184	0.128	0.293
Amylase	−0.141	0.248	−0.166	0.163
Lipase	0.003	0.978	−0.067	0.582
Cholesterol	0.149	0.222	−0.091	0.458
HDL-C	−0.046	0.709	−0.140	0.252
LDL-C	0.024	0.844	−0.043	0.726
Triglycerides	0.458	<0.001 *	−0.010	0.933

* *p* < 0.05 shows statistical significance. Abbreviations: ALP, alkaline phosphatase; ALT, alanine aminotransferase; AST, aspartate aminotransferase; GGT, gamma-glutamyl transferase, HbA1-c, hemoglobin A1c; HDL-C, high-density lipoprotein cholesterol; LDL-C, low-density lipoprotein cholesterol; LFF, liver fat fraction; PFF, pancreatic fat fraction; r, correlation coefficient.

**Table 4:** Independent predictors of liver and pancreatic fat fractions.

Variables	Univariable Regression			Multivariable Regression		
	ꞵ ± SE	95% CI	*p*	ꞵ ± SE	95% CI	*p*
log(LFF)						
Male gender	−0.18 ± 0.06	(−0.29)–(−0.06)	0.003 *	−0.21 ± 0.05	(−0.31)–(−0.10)	0.007 *
BMI	0.09 ± 0.04	0.02–0.17	0.017 *	-	-	-
Waist circumference	0.06 ± 0.02	0.01–0.11	0.009 *	0.05 ± 0.02	0.01–0.09	0.033 *
Insulin use	−0.17 ± 0.06	(−0.29)–(−0.05)	0.007 *	−0.16 ± 0.05	(−0.27)–(−0.06)	0.010 *
log(ALT)	0.71 ± 0.15	0.41–1.01	<0.001 *	0.67 ± 0.14	0.40–0.94	<0.001 *
log(Triglyceride)	0.50 ± 0.13	0.24–0.77	<0.001 *	0.36 ± 0.12	0.12–0.59	0.003 *
				Adjusted R^2^ = 0.33, *p* < 0.001 *		
log(PFF)						
Atorvastatin	0.19 ± 0.07	0.05–0.33	0.001 *	0.17 ± 0.06	0.06–0.28	0.011 *
Rosuvastatin	−0.18 ± 0.06	(−0.30)–(−0.06)	0.010 *	−0.16 ± 0.07	(−0.30)–(−0.02)	0.025 *
Pitavastatin	0.21 ± 0.08	0.05–0.37	0.008 *	0.19 ± 0.07	0.05–0.33	0.008 *
log(ALP)	0.44 ± 0.16	0.11–0.76	<0.001 *	0.33 ± 0.16	0.01–0.64	0.046 *
				Adjusted R^2^ = 0.21, *p* < 0.001 *		

Prior to the regression analysis, logarithmic transformation was applied to numerical variables that did not exhibit a normal distribution. In all multivariable regression models, the effects of age, alcohol use, duration of diabetes, and comorbidities were adjusted. Additionally, in the regression model created for log(PFF), the effects of BMI, waist circumference, and insulin use were adjusted. * *p* < 0.05 shows statistical significance. Abbreviations: see Table 1, Table 2 and Table 3; ꞵ, regression coefficient; CI, confidence interval; SE, standard error.

## Data Availability

The data that support the findings of this study are available on request from the corresponding author.

## Supplementary Materials

The following supporting information can be downloaded at: https://www.mdpi.com/article/10.3390/diagnostics15040426/s1, Table S1: Distribution of demographic and clinical characteristics among type 2 diabetes mellitus patients according to types of statin used.
