# Supplementary material for: The Association of Statin Therapy with Liver and Pancreatic Fat Fraction in Type 2 Diabetes Mellitus
Source: Diagnostics (Basel). 2025 Feb 10;15(4):426. doi: 10.3390/diagnostics15040426 (PMC11854770; doi:10.3390/diagnostics15040426)
Supplement: Supplementary file 1 [file diagnostics-15-00426-s001.zip › diagnostics-3418168-supplementary.pdf]

Supplementary Table SI. Distribution of demographic and clinical characteristics among type 2 diabetes mellitus patients according to types of statin used.

Variables	Statin				p
	No<br>n = 70	Atorvastatin<br>n = 53	Rosuvastatin<br>n = 8	Pitavastatin<br>n = 9	
Female gender, n (%)	40 (57.1)	25 (47.2)	4 (50.0)	8 (88.9)	0.127
Age, years	54.2 ± 8.7	57.7 ± 7.7	57.6 ± 5.7	55.2 ± 8.3	0.117
BMI, kg/m^2	30.9 ± 8.0	31.5 ± 6.6	30.0 ± 4.0	30.9 ± 12.8	0.949
Waist circumference, cm	102.9 ± 11.4	103.6 ± 13.6	101.6 ± 11.1	107.3 ± 9.5	0.741
Alcohol use, n (%)	12 (17.1)	7 (13.2)	-	-	0.497
Duration of diabetes, years	6.0 (2.2-12.0)	8.0 (5.0-13.0)	10.0 (3.5-17.0)	7.0 (5.0-25.0)	0.276
Comorbidities, n (%)					
Hypertension	26 (37.1)	30 (56.6)	5 (62.5)	4 (44.4)	0.140
Cardiac disease	5 (7.1)	8 (15.1)	1 (12.5)	-	0.359
Thyroid diseases	7 (10.0)	8 (15.1)	-	1 (11.1)	0.598
Hyperlipidemia	**22 (31.4)**†	50 (94.3)	8 (100.0)	8 (88.9)	<0.001*
Anti-diabetic drugs					
Only OADs	46 (65.7)	33 (62.3)	6 (75.0)	7 (77.8)	
Only insulin	1 (1.4)	3 (5.7)	-	-	0.820
Combined therapy	23 (32.9)	17 (32.1)	2 (25.0)	2 (22.2)	
Hepatosteatosis, n (%)	64 (91.4)	43 (81.1)	7 (87.5)	8 (88.9)	0.410
Liver fat fraction, %	9.0 (5.1-14.7)	8.3 (3.8-13.0)	8.1 (5.1-16.1)	14.8 (7.8-20.1)	0.445
Pancreatic fat fraction, %	**6.2 (4.2-9.3)**†	8.7 (5.5-14.9)	**3.2 (2.6-6.6)**†	9.2 (3.5-10.2)	0.004*
Laboratory findings					
Glucose, mg/dL	142.0 (121.0-184.5)	131.0 (104.0-170.0)	113.0 (101.5-142.0)	146.0 (103.0-159.0)	0.176
Leukocytes, ×10^3/mm^3	7.9 ± 2.5	7.9 ± 1.7	7.4 ± 1.4	7.8 ± 3.4	0.946
Platelets, ×10^3/mm^3	269.1 ± 58.0	269.2 ± 59.4	287.9 ± 58.8	289.3 ± 61.1	0.652
HbA1-c, %	8.3 ± 2.3	7.7 ± 1.7	7.7 ± 1.4	7.2 ± 1.5	0.186
Urea, mg/dL	29.0 (25.0-32.0)	30.0 (25.0-36.0)	31.0 (26.0-38.0)	26.0 (24.0-34.0)	0.534
Creatinine, mg/dL	0.7 (0.6-0.8)	0.8 (0.7-0.9)	0.8 (0.8-1.0)	0.8 (0.6-0.8)	0.095
ALT, U/L	18.1 (14.0-23.0)	21.0 (15.0-26.0)	16.3 (12.8-18.8)	15.0 (13.2-22.0)	0.204
AST, U/L	16.0 (14.0-18.8)	17.0 (15.0-20.9)	19.0 (16.6-20.2)	17.0 (14.0-19.0)	0.190
ALP, U/L	77.5 (64.0-96.0)	81.0 (71.0-100.0)	71.5 (65.8-77.5)	70.0 (63.0-122.0)	0.287
GGT, U/L	23.0 (15.2-30.8)	25.0 (17.0-37.0)	23.5 (15.5-34.2)	22.0 (14.0-26.0)	0.465
Amylase, U/L	54.0 (41.2-82.8)	61.0 (55.0-74.0)	67.0 (54.2-73.2)	66.0 (54.0-75.0)	0.491
Lipase, U/L	32.5 (23.0-41.5)	28.0 (24.0-37.7)	37.0 (33.8-60.5)	26.7 (20.0-39.0)	0.146
Cholesterol, mg/dL	**221.6 ± 66.5**†	181.0 ± 42.3	188.9 ± 49.4	180.3 ± 40.6	0.026*
HDL-C, mg/dL	48.4 ± 12.8	46.0 ± 13.0	48.4 ± 13.1	51.0 ± 12.5	0.629
LDL-C, mg/dL	**119.5 (96.2-142.8)**†	102.0 (78.0-130.0)	84.0 (77.5-123.5)	100.0 (88.0-151.0)	0.044*
Triglycerides, mg/dL	164.0 (101.2-212.2)	126.0 (98.0-180.0)	233.0 (136.8-246.2)	130.0 (90.0-144.0)	0.067

Numerical variables were shown as mean ± standard deviation or median (IQR). Categorical variables were shown as numbers (%). * P <0.05 shows statistical significance. † Indicates groups that showed differences in the post-hoc analysis. Abbreviations: ALP, alkaline phosphatase; ALT, alanine aminotransferase; AST, aspartate aminotransferase; BMI, body mass index; GGT, gamma glutamyl transferase, HbA1-c, hemoglobin A1c; OAD, oral anti-diabetic drugs; HDL-C, high density lipoprotein cholesterol; LDL-C, low density lipoprotein cholesterol.
